# Extended use of dual antiplatelet therapy among older adults with acute coronary syndromes and associated variables: a cohort study

**DOI:** 10.1186/s12959-023-00476-5

**Published:** 2023-03-21

**Authors:** Albert Ariza-Solé, Gemma Mateus-Porta, Francesc Formiga, Sergio Garcia-Blas, Clara Bonanad, Iván Núñez-Gil, Carlos Vergara-Uzcategui, Pablo Díez-Villanueva, Jordi Bañeras, Clara Badia-Molins, Jaime Aboal, José Carreras-Mora, Ana Gabaldón-Pérez, José Antonio Parada-Barcia, Manuel Martínez-Sellés, Josep Comín-Colet, Sergio Raposeiras-Roubin

**Affiliations:** 1 Cardiology Department, Bioheart Grup de Malalties Cardiovasculars, Hospital Universitari de Bellvitge, Institut d’Investigació Biomèdica de Bellvitge—IDIBELL, L’Hospitalet de Llobregat, Feixa Llarga s/n. 08907, Barcelona, Spain; 2 Geriatrics Unit. Internal Medicine Department, Hospital Universitari de Bellvitge. L’Hospitalet de Llobregat, Barcelona, Spain; 3 Cardiology Department, Department of Medicine, Hospital Clínico Universitario de Valencia, INCLIVA Biomedical Research Institute, University of Valencia, Valencia, Spain; 4 Cardiology Department, Hospital Clínico San Carlos, Madrid, Spain; 5 Cardiology Department, Hospital Universitario de la Princesa, Madrid, Spain; 6 Cardiology Department, Hospital Universitari Vall d’Hebrón, Barcelona, Spain; 7 Cardiology Department, Hospital Josep Trueta, Girona, Spain; 8 Cardiology Department, Hospital del Mar, Barcelona, Spain; 9 Cardiology Department, Hospital Universitario Álvaro Cunqueiro de Vigo, Pontevedra, Spain; 10 Cardiology Department, Hospital Universitario Gregorio Marañón, CIBERCV. Universidad Europea. Universidad Complutense, Madrid, Spain

**Keywords:** Elderly, Acute coronary syndromes, Dual antiplatelet therapy, Bleeding risk

## Abstract

**Background:**

Current guidelines recommend extending the use of dual antiplatelet therapy (DAPT) beyond 1 year in patients with an acute coronary syndrome (ACS) and a high risk of ischaemia and low risk of bleeding. No data exist about the implementation of this strategy in older adults from routine clinical practice.

**Methods:**

We conducted a Spanish multicentre, retrospective, observational registry-based study that included patients with ACS but no thrombotic or bleeding events during the first year of DAPT after discharge and no indication for oral anticoagulants. High bleeding risk was defined according to the Academic Research Consortium definition. We assessed the proportion of cases of extended DAPT among patients 65 ≥ years that went beyond 1 year after hospitalisation for ACS and the variables associated with the strategy.

**Results:**

We found that 48.1% (928/1,928) of patients were aged ≥ 65 years. DAPT was continued beyond 1 year in 32.1% (298/928) of patients ≥ 65; which was a similar proportion as with their younger counterparts. There was no significant correlation between a high bleeding risk and DAPT duration. Contrastingly, there was a strong correlation between the extent of coronary disease and DAPT duration (p < 0.001). Other variables associated with extended DAPT were a higher left ventricle ejection fraction, a history of heart failure and a prior stent thrombosis.

**Conclusion:**

There was no correlation between age and extended use of DAPT beyond 1 year in older patients with ACS. DAPT was extended in about one-third of patients ≥ 65 years. The severity of the coronary disease, prior heart failure, left ventricle ejection fraction and prior stent thrombosis all correlated with extended DAPT.

**Supplementary Information:**

The online version contains supplementary material available at 10.1186/s12959-023-00476-5.

## Introduction

Dual antiplatelet therapy (DAPT) is one of the main therapeutic tools used in patients with an acute coronary syndrome (ACS) undergoing percutaneous coronary intervention (PCI). Based on the results of the DAPT [1] and PEGASUS TIMI 54^2^ trials, current guidelines recommend extending DAPT beyond 1 year for ACS patients with a high ischaemic risk and without a high bleeding risk [2]. There is scant information in the literature about the recommendation’s applicability to routine clinical practice. The DAISY registry [3] recently assessed a series of 1,967 patients who were free from any thrombotic or bleeding events 1 year after discharge for an ACS admission. DAPT was continued beyond 1 year in approximately one-third of potential candidates. Predictors of DAPT prolongation were a history of PCI, stent thrombosis, coronary artery disease (CAD) complexity, reinfarction and clopidogrel use.

The progressive ageing of Western populations and the greater incidence of ACS among older patients are driving a significant increase in the number of older adults admitted with a diagnosis of ACS [4]. These patients have a higher risk of bleeding and thrombotic events [5] and are often excluded from clinical trials [6]. Most elderly patients are managed conservatively in routine clinical practice because of the risk of bleeding [7]. In this context, clopidogrel is generally prescribed in preference to ticagrelor or prasugrel [8]. There are no data available about the real-world implementation of extended DAPT strategies, and their associated predictors, in older adults with ACS.

The aim of this cohort study based on the DAISY database was to assess the proportion of potential candidates ≥ 65 years who received extended DAPT (i.e., for more than 1 year) and identify the variables associated with extended treatment.

## Methods

The DAISY registry [3] was a multicentre retrospective study conducted at 10 Spanish centres between January 2017 and December 2018 that enrolled consecutive patients with ACS surviving to hospital discharge who were free from ischaemic or bleeding events throughout the first year after being discharged. This study was endorsed by the *Geriatric Cardiology Section* and the *Association of Ischaemic Heart Disease and Acute Cardiac Care* of the *Spanish Society of Cardiology*. A detailed description of the study design has been published previously [3]. The main aim of this subanalysis was to assess the clinical characteristics, management and variables associated with extended DAPT (i.e., for more than 1 year) among patients ≥ 65 years in the DAISY registry.

### Population

The DAISY registry included patients who were candidates for DAPT prolongation 1 year after hospitalisation for ACS. Specifically, the inclusion criteria were: (a) obstructive coronary artery disease (defined as 50% stenosis of the left main coronary artery or 70% in other arteries) diagnosed by coronary angiography; (b) PCI performance at admission; (c) DAPT at discharge; and (d) survival 1 year after discharge. The exclusion criteria were: (a) indication for oral anticoagulants at discharge or within the first year; and (b) any ischaemic or bleeding events or the need for a blood transfusion within 1 year after being discharged. For the purpose of this subanalysis, we focused on patients aged ≥ 65 years.

### Data collection and definitions

Data was collected by trained, local investigators using standardised electronic forms. Demographic, clinical, laboratory and echocardiographic data, the severity and extent of the coronary disease, and treatment at discharge were recorded for each patient. A CAD extension variable was calculated based on the presence of significant stenosis in the left main coronary and proximal left anterior descending arteries and whether the patient had multivessel disease (at least two coronary territories); each variable was assigned one point. The criteria applied in the PEGASUS clinical trial [9] were also assessed for each patient (> 65 years, second infarction, diabetes, multivessel disease or kidney failure). A high bleeding risk was defined as the presence of at least one major high-risk criterion or two minor criteria according to the Academic Research Consortium (ARC-HBR) [2]. The moment of DAPT discontinuation was recorded if it occurred within the maximum follow-up available for each patient.

### Clinical outcomes

The main outcome measured was discontinuation of DAPT 1 year after admission for ACS. Given that medical visits are not always at exactly 12 months in routine clinical practice, DAPT discontinuation was considered at 1 year if the treatment was stopped within 14 months (425 days). Clinical follow-up was performed by reviewing the subjects’ medical records.

### Ethics

This observational study was performed in accordance with the ethical principles set out in the Declaration of Helsinki. Confidential patient information was protected according to national regulations. The protocol was revised and approved by the Clinical Research Ethics Committee of Bellvitge University Hospital (IRB00005523).

### Statistical analysis

The normal distribution of variables was analysed using the Shapiro–Wilk test. Quantitative variables are expressed as means and standard deviation. Non-normally distributed variables are expressed as medians and interquartile ranges. Categorical variables are expressed as numbers and percentages.

Baseline characteristics and clinical management were compared across age groups (< 65 vs. ≥ 65 years). The association between categorical variables was analysed with the chi-square test, applying a continuity correction factor when indicated. We used Student’s t-test to compare the quantitative variables according to the two age categories. We also compared three different elderly subgroups with patients aged 65–75, 75–85 and > 85 years, using an ANOVA test to compare the quantitative variables for the four age categories.

Variables associated with 1year DAPT discontinuation among patients aged ≥ 65 were assessed through a binary logistic regression model. Those variables that showed an association with extended DAPT (p < 0.2) in the univariate analysis were included in the multivariate analysis. Results are expressed as odds ratio, 95% confidence intervals and p-values. Analyses were performed with the aid of SPSS Statistics, Version 21 (SPSS Inc., USA).

## Results

We determined that 48.1% (928/1,928) of all patients who received DAPT for more than 1 year were ≥ 65 years. There were more females than males among the older adults and they had a higher prevalence of comorbidities, such as hypertension, diabetes, prior stroke, peripheral artery disease and prior heart failure (p < 0.001 for all comparisons, see Table 1). This group also had a higher proportion of prior neoplasms or bleeding events, more PEGASUS criteria and a higher bleeding risk according to ARC criteria. Furthermore, patients ≥ 65 years had a higher overall risk profile, with a greater proportion of Killip class ≥ II at admission, poorer renal function, more extensive and complex CAD, and a lower probability of undergoing complete revascularisation (Table 1).

**Table 1:** Clinical characteristics and management by age

Variable	Whole series (n = 1,928)	Patients aged ≥ 65 (n = 928)	Patients aged < 65 (n = 1,000)	p-value (≥ 65 vs. < 65)
Age (years)	64.5 (13)	75.7 (7)	54.1 (7)	< 0.001
Male	1509 (78.3)	649 (69.9)	860 (86)	< 0.001
Hypertension	1113 (57.9)	657 (70.8)	456 (45.6)	< 0.001
Diabetes	526 (27.4)	332 (35.8)	194 (19.4)	< 0.001
Dyslipidaemia	1055 (54.8)	539 (58.1)	516 (51.6)	0.004
Active smoker	736 (38.3)	149 (16.1)	587 (58.7)	< 0.001
Peripheral artery disease	148 (7.7)	103 (11.1)	45 (4.5)	< 0.001
Prior stroke	89 (4.7)	66 (7.1)	23 (2.3)	< 0.001
Prior MI^a^	915 (47.5)	453 (48.8)	462 (46.2)	0.251
Prior PCI^b^	273 (14.2)	173 (18.6)	100 (10)	< 0.001
Prior stent thrombosis	33 (1.7)	18 (1.9)	15 (1.5)	0.457
Prior heart failure	49 (2.6)	39 (4.2)	10 (1)	< 0.001
Prior bleeding event	31 (1.6)	21 (2.3)	10 (1)	0.028
Active neoplasm	61 (3.2)	50 (5.4)	11 (1.1)	< 0.001
Total PEGASUS criteria	1.9 (1)	2.7 (1)	1.1 (1)	< 0.001
High bleeding risk criteria (%)	708 (36.7)	516 (55.6)	192 (19.2)	< 0.001
Creatinine clearance (mL/min)	88 (38)	67 (25)	110 (37)	< 0.001
Clinical presentation ACS^c^				< 0.001
• Unstable angina	244 (12.7)	156 (16.8)	88 (8.8)	
• NSTEMI^d^	728 (38)	378 (40.7)	350 (35)	
• STEMI^e^	946 (49.3)	388 (41.8)	558 (55.8)	
Killip class on admission ≥ II	224 (11.7)	136 (14.7)	88 (8.8)	< 0.001
Left main or multivessel disease	1280 (66.5)	676 (72.8)	604 (60.4)	< 0.001
Number of stents	1.5 (1.3)	1.5 (1)	1.4 (1)	0.071
Total length stents (mm)	30.2 (20)	30.7 (20)	29.9 (19)	0.372
Complete revascularization	417 (21.9)	228 (24.6)	189 (18.9)	0.002
Left ventricle ejection fraction (%)	55 (10)	55 (10)	55 (10)	0.872
In-hospital reinfarction	21 (1.1)	8 (0.9)	13 (1.3)	0.355
In-hospital bleeding	22 (1.1)	12 (1.3)	10 (1)	0.545
P2Y12 inhibitor at discharge				< 0.001
Clopidogrel	577 (30.1)	401 (43.2)	176 (17.6)	
Prasugrel	212 (11.1)	54 (5.8)	158 (15.8)	
Ticagrelor	1129 (58.9)	469 (50.5)	660 (66)	
DAPT^f^ discontinuatiom				0.201
• Before one year	130 (6.7)	72 (7.7)	58 (5.8)	
• At one year	1183 (61.4)	558 (60.1)	625 (62.5)	
• Beyond one year	615 (31.9)	298 (32.1)	317 (31.7)	

Categorical variables are expressed as n (%). Quantitative variables are expressed as mean (SD).

(a) MI: myocardial infarction; (b) PCI: percutaneous coronary intervention; (c) ACS: acute coronary syndrome; (d) NSTEMI: non-ST-segment elevation myocardial infarction; (e) STEMI: ST-segment elevation myocardial infarction; (f) DAPT: dual antiplatelet therapy

**Fig. 1:**
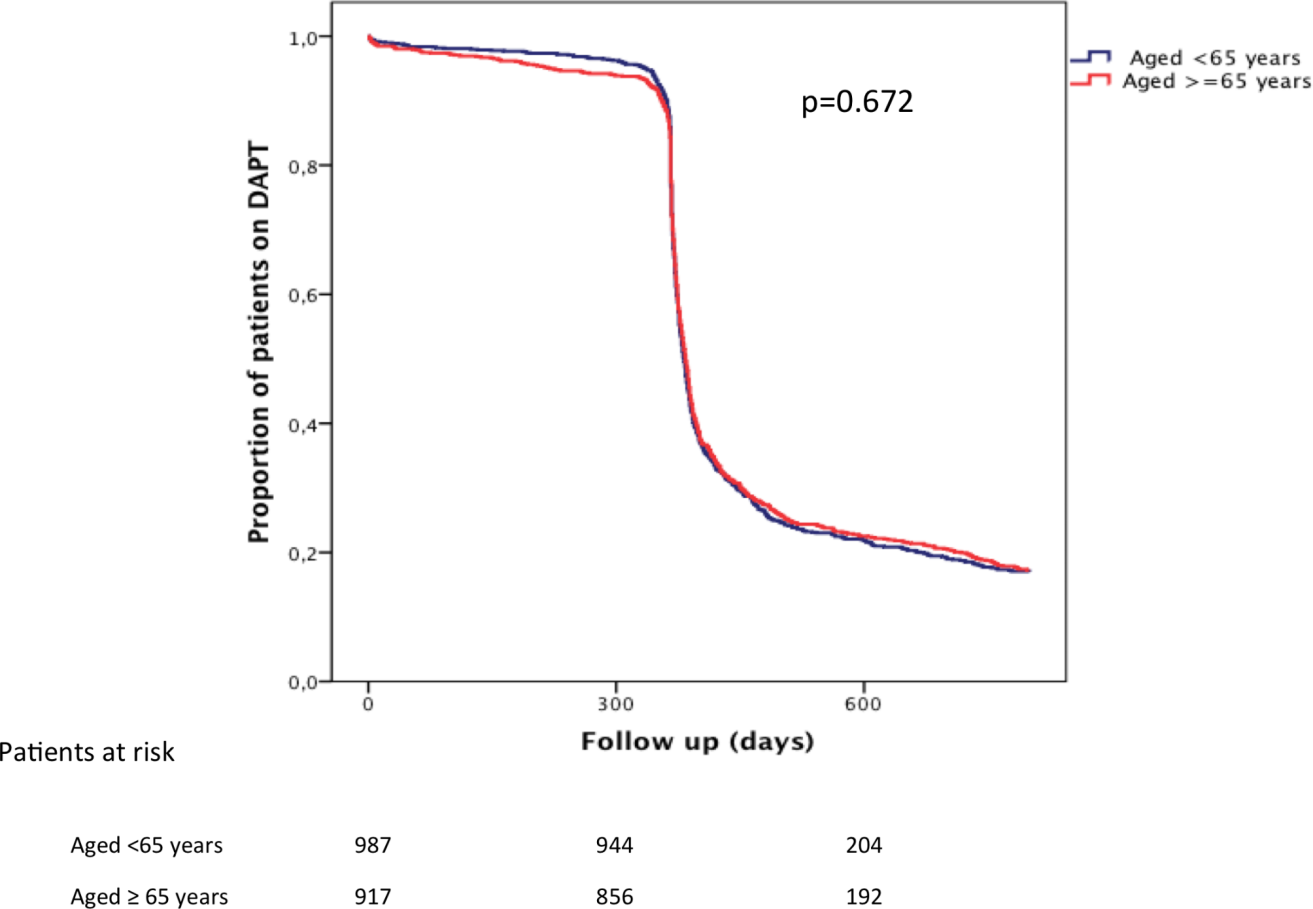
Percentage of patients remaining on dual antiplatelet therapy by age group

Although ticagrelor was the most prescribed P2Y12 inhibitor at discharge in both groups, the proportion was significantly lower among patients ≥ 65 years (50.5% vs. 66%, p < 0.001, Table 1). There were no significant differences regarding extended DAPT (beyond 1 year) between the two groups (32.1% among those ≥ 65 vs. 31.7% for subjects < 65, p = 0.201, Table 1). Overall, the length of DAPT was similar regardless of age (p = 0.672, Fig. 1).

### Clinical characteristics associated with extended DAPT

There was a significant correlation between extended DAPT and higher rates of dyslipidaemia, diabetes and prior heart failure, PCI or stent thrombosis. The most significant correlations were observed for prior heart failure, prior stent thrombosis and CAD complexity (p < 0.001 in all cases, Table 2). No significant differences were observed regarding age, gender and number of underlying comorbidities (Table 2).

**Table 2:** Variables associated with extended DAPT among patients ≥ 65 years

	Univariate analysis		Multivariate analysis	
Variable	Odds ratio (95% CI)	p-value	Odds ratio (95% CI)	p-value
Age*	1.01 (0.99–1.03)	0.289		
Male	0.87 (0.64–1.18)	0.371		
Hypertension	1.36 (0.99–1.87)	0.630		
Diabetes	1.22 (1.05–1.40)	0.008		
Dyslipidaemia	1.41 (1.06–1.88)	0.019		
Active smoker	0.95 (0.78–1.14)	0.946		
Peripheral artery disease	1.43 (0.93–2.19)	0.105		
Prior stroke	1.48 (0.88–2.49)	0.141		
Prior MI^a^	0.81 (0.61–1.07)	0.140		
Prior PCI^b^	1.81 (1.28–2.56)	0.001		
Prior stent thrombosis	7.68 (2.50–23.5)	< 0.001	6.45 (1.72–24.1)	0.006
Prior heart failure	3.58 (1.77–7.27)	< 0.001	3.44 (1.58–7.48)	0.002
Prior bleeding event	0.58 (0.28–1.18)	0.841		
Active neoplasm	0.70 (0.29–1.68)	0.129		
Total PEGASUS criteria*	1.22 (1.07–1.39)	0.003		
High bleeding risk criteria	1.18 (0.89–1.57)	0.239		
PRECISE DAPT scoring*	1.02 (1.01–1.04)	0.023		
Creatinine clearance (mL/min)*	0.99 (0.99–1.01)	0.253		
Left main or multivessel disease	1.99 (1.41–2.82)	< 0.001	1.95 (1.36–2.79)	< 0.001
Number of stents	0.97 (0.86–1.09)	0.601		
Total length of stents	1.00 (0.99–1.01)	0.290		
Left ventricle ejection fraction*	1.02 (1.00-1.03)	0.054	1.03 (1.01–1.04)	0.002
Killip class at admission ≥ II	1.49 (1.02–2.20)	0.041		
In-hospital reinfarction	3.55 (0.84–14.9)	0.084		

(a) MI: myocardial infarction; (b) PCI: percutaneous coronary intervention

*For quantitative variables, OR reflects the increased probability of extended DAPT as follows: age: per year of increase; PEGASUS criteria: for each additional PEGASUS criteria; PRECISE DAPT scoring: for each integer of increase of PRECISE DAPT value: left ventricle ejection fraction: for each % of increase

### Duration of DAPT depending on antiplatelet therapy at discharge, bleeding risk and extent of coronary disease

The most prescribed P2Y12 at discharge for patients ≥ 65 was ticagrelor (50.5%), followed by clopidogrel (43.2%). There was no significant relationship between the P2Y12 inhibitor prescribed at discharge and DAPT duration.

**Fig. 2:**
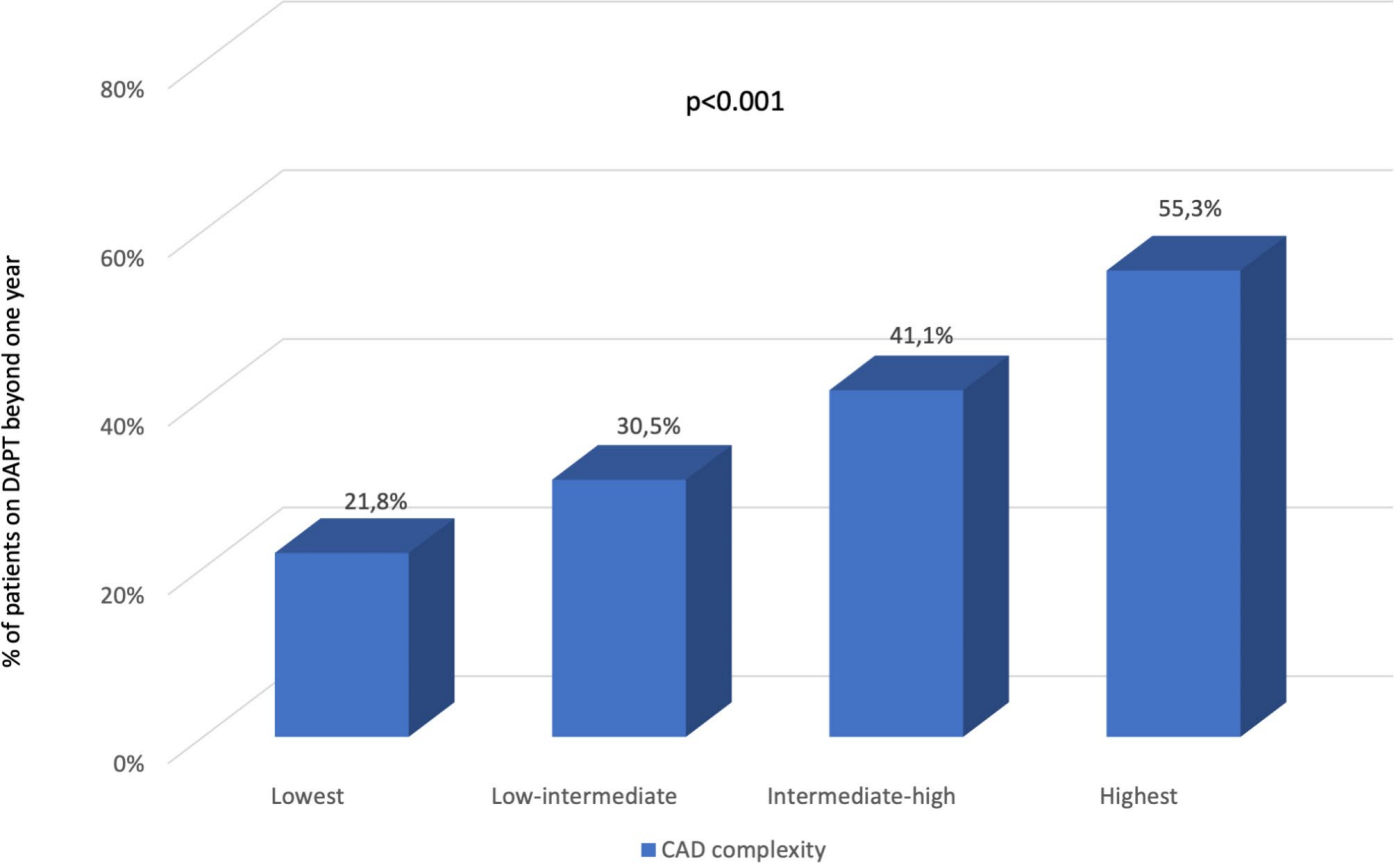
Percentage of patients who received extended dual antiplatelet therapy according to the complexity of their coronary artery disease. Coronary artery disease complexity was calculated based on the presence of significant stenosis in the left main and proximal left anterior descending coronary arteries and whether the patient had multivessel disease (at least two coronary territories), assigning one point in each case

Most patients aged ≥ 65 had a high bleeding risk (55.6%). However, there was no signification correlation between ARC-HBR score and DAPT duration (p = 0.239, Table 2). The proportion of patients requiring extended DAPT (beyond 1 year) progressively increased along with the severity of coronary artery disease (CAD) (p < 0.001, Fig. 2).

### Independent variables associated with extended DAPT

The severity of CAD showed the strongest association with extended DAPT (p < 0.001, Table 2). The other variables showing an independent association with extended DAPT in the final model were a left ventricle ejection fraction (p = 0.002), prior heart failure (p = 0.002) and prior stent thrombosis (p = 0.006) (Table 2).

### Age subgroups among patients ≥ 65 years

An additional analysis was conducted among patients ≥ 65 years by comparing three different subgroups (65–75, 75–85 and > 85 years). We evidenced a significant linear trend between increasing age subgroups and a lower proportion of males and higher rates of hypertension and prior stroke (Supplementary Table 1). Similarly, older patients had a progressively higher number of PEGASUS criteria, greater bleeding risk, poorer renal function, more complex and extensive CAD, higher proportion of Killip class ≥ II at admission, and poorer left ventricle ejection fraction. Patients from the oldest groups were mostly treated with clopidogrel and less likely to receive ticagrelor, but there were no significant differences regarding the proportion of patients who required extended DAPT. (Supplementary Table 1). Overall, we did not find any significant differences in terms of DAPT duration across the elderly subgroups (p = 0.879, Supplementary Fig. 1).

## Discussion

The main findings of our study are: (a) in this Spanish retrospective cohort study, the use of DAPT was extended beyond 1 year after admission in roughly one-third of ACS patients ≥ 65 years; (b) there was no significant correlation between age and need for extended DAPT in this age range; (c) most patients ≥ 65 years had a high bleeding risk according to ARC criteria, but this did not correlate to DAPT duration; and (d) independent variables associated with extended DAPT among patients aged ≥ 65 were the severity of CAD, a history of prior heart failure, left ventricle ejection fraction and prior stent thrombosis.

The DAPT trial [1] reported a reduced rate of ischaemic events in patients whose DAPT was extended beyond 1 year after PCI compared to when it was discontinued at 12 months. The PEGASUS-TIMI 54 study [9] randomised patients with prior myocardial infarction and at least one other risk factor to aspirin or aspirin plus ticagrelor at doses of 60 mg or 90 mg once daily. The authors observed a significant reduction in the risk of ischaemic events for the patients given ticagrelor. Based on the results of these two trials, current guidelines [2] recommend extending DAPT beyond 1 year in patients with a high risk of ischaemic events and no significant risk of major bleeding. However, there is very little information about the implementation of this strategy in routine clinical practice, especially in older patients. This is important because older patients pose a higher risk for bleeding [5] and ischaemic events and prolonged DAPT strategies in these patients may have a significantly different impact [10]. In addition, older patients are often excluded from clinical trials. Interestingly, routine clinical practice suggests that patients with ACS who meet more PEGASUS criteria will have poorer outcomes [11].

There is still some controversy about which patients should receive extended DAPT in routine clinical practice and how age affects this decision. The EPICOR Asia study [12] included patients with NSTEMI. Most patients (90.8%) were receiving DAPT at discharge. At 2-year follow-up, this proportion was 60%. Patients who stopped DAPT ≤ 12 months post-discharge tended to be older, female, have prior cardiovascular disease and renal dysfunction. Bardaji et al. [13] assessed a series of 782 patients with ACS enrolled in the Spanish cohort of the EPICOR study. More than half the patients (53.1%) remained on DAPT at 2 years. Patients > 65 years with diabetes were more likely to continue with DAPT up to 2 years after discharge. The PARIS [14] registry showed that 37.2% of patients undergoing PCI between 2009 and 2010 were on DAPT at 2 years. In the START ANTIPLATELET study [14], the decision to prolong DAPT beyond 1 year was taken in 13% of patients with ACS. Variables associated with DAPT continuation were a new cardiovascular event after the index admission event, no bleeding complications and no anaemia at 1-year follow-up; other associated variables were kidney failure and peripheral artery disease. Notably, the decision to extend DAPT did not correlate with age.

Data from our series revealed that DAPT was extended beyond 1 year in around one in three patients ≥ 65 years. The low percentage of extended DAPT in the START ANTIPLATELET study [15] might be because it was conducted before ticagrelor use was approved for more than 1 year after ACS in Italy. The percentage of extended DAPT in our study was even lower than the EPICOR Asia study. It is important to note that the DAISY registry was the only study that excluded patients with ischaemic or bleeding events in the first year after discharge. This may change the clinical profile of patients and their indications for extended DAPT.

Interestingly, there was no significant correlation between age and DAPT extension in our study. Adherence to recommendations is usually lower in these patients and the antithrombotic approach is conservative in most series, probably due to a higher burden of comorbidities [16]. Older patients treated with potent antiplatelet drugs in routine clinical practice are less frail and have fewer comorbidities and other geriatric syndromes [17]. Current recommendations emphasise the need to adapt the intensity and duration of antiplatelet strategies to each patient’s risk profile. The *American Geriatrics Society BEERS Criteria®* recommend the careful use of potent antiplatelet drugs in elderly patients because of the risk of bleeding [18]. In our study, as we only included patients with no bleeding or ischaemic events in the first year and no indication for oral anticoagulation, it may result in a select series of patients with a lower comorbidity burden and less frailty. In fact, the rate of relevant comorbidities in our study sample, such as diabetes, stroke, kidney failure, anaemia or peripheral artery disease, was significantly lower compared to other important series of patients with ACS [19].

The EPICOR Asia study [12] included all patients surviving to discharge and there was a significant association between age and a shorter DAPT duration. By contrast, in the Spanish cohort of the EPICOR study [13], patients ≥ 65 years were more likely to still be on DAPT at 2 years. The lack of association between age and DAPT duration in the START ANTIPLATELET study [15] might be down to other factors, such as logistical or economic conditions. Globally, it remains a question open to assessment in larger series, as other variables that have not been assessed yet might play a significant role when deciding whether to extend DAPT.

There was no significant correlation between bleeding risk and DAPT duration in the older patients from our series. On the other hand, there was a strong association between the severity of coronary disease and a longer DAPT strategy. The percentage of patients with extended DAPT increased progressively along with the severity of CAD. In our opinion, the fear of ischaemic events in patients with complex CAD [20] may be more important for physicians than the perceived risk of bleeding when deciding the optimal antithrombotic approach in routine clinical practice. The association between prior stent thrombosis and extended DAPT also supports this hypothesis.

This study has certain limitations, such as its observational retrospective design. Therefore, we cannot rule out the presence of residual confounding. As we only included patients without any ischaemic or bleeding events in the first year after discharge, this implies a significant selection bias. However, this selection allowed us to obtain a reliable picture of the current implementation of extended DAPT among real candidates for longer DAPT regimens and therefore assess the real adherence to current recommendations. Our entire cohort was from a single country, so our findings should be validated in other geographic settings. Furthermore, the lack of data on clinical outcomes (cardiovascular and bleeding events), as well as on frailty, disability and other ageing related variables (which are closely related to management [21, 22] and prognosis [23–25]) are major limitations of our study. Finally, defining CAD complexity through a validated score might have provided additional information.

Nevertheless, despite these limitations, this study provides novel and relevant information about extending DAPT beyond 1 year in older patients with ACS and associated variables in routine clinical practice. Future studies should focus on assessing the clinical outcomes in function of DAPT duration in older patients with ACS. Improving clinical management and outcomes in these high risk patients could lead to significant clinical, economic and social benefits.

## Conclusion

DAPT was extended beyond 1 year after discharge for ACS in approximately one-third of patients aged ≥ 65 years. There was no significant correlation between neither age nor a high bleeding risk and the need to extend DAPT among these patients. Contrastingly, we observed a strong association between extended DAPT and CAD severity. Other variables that correlated with extended DAPT were prior heart failure, left ventricle ejection fraction and prior stent thrombosis.

## Electronic supplementary material

Below is the link to the electronic supplementary material.

Supplementary Material 1

Supplementary Material 2

## Data Availability

The datasets used and/or analysed during the study are available from the corresponding author on reasonable request.

## Abbreviations

DAPT: Dual antiplatelet therapy
ACS: Acute coronary syndrome
PCI: Percutaneous coronary intervention
ROC: Receiver operating characteristic
NSTEMI: curves; non-ST-segment elevation myocardial infarction
CAD: Coronary artery disease
