# Supplementary material for: Extended use of dual antiplatelet therapy among older adults with acute coronary syndromes and associated variables: a cohort study
Source: Thromb J. 2023 Mar 21;21:32. doi: 10.1186/s12959-023-00476-5 (PMC10031931; doi:10.1186/s12959-023-00476-5)
Supplement: Supplementary file 1 — Supplementary Material 1 [file 12959_2023_476_MOESM1_ESM.docx]

Supplementary Table 1. Clinical characteristics, management and outcomes by age subgroup among patients ≥ 65 years.

| Variable | Patients 65–75 years (n=455) | Patients 75–85 years (n=342) | Patients ≥ 85 years (n=131) | p-value (for linear trend) |
| --- | --- | --- | --- | --- |
| Age (years) | 69.5 (3) | 79.4 (3) | 87.6 (3) | <0.001 |
| Male | 358 (79) | 226 (66.1) | 65 (49.6) | <0.001 |
| Hypertension | 296 (65.3) | 257 (75.4) | 104 (79.4) | <0.001 |
| Diabetes | 152 (33.5) | 141 (41.4) | 39 (29.8) | 0.789 |
| Dyslipidaemia | 265 (58.4) | 206 (60.4) | 68 (51.9) | 0.402 |
| Active smoker | 116 (25.5) | 27 (7.9) | 6 (4.6) | <0.001 |
| Peripheral artery disease | 43 (9.5) | 46 (13.5) | 14 (10.7) | 0.319 |
| Prior stroke | 21 (4.6) | 34 (10) | 11 (8.4) | 0.018 |
| Prior MI^a^ | 215 (47.3) | 172 (50.3) | 66 (50.4) | 0.401 |
| Prior PCI^b^ | 77 (16.9) | 73 (21.3) | 23 (17.6) | 0.450 |
| Prior stent thrombosis | 9 (2) | 7 (2) | 2 (1.5) | 0.812 |
| Prior heart failure | 15 (3.3) | 16 (4.7) | 8 (6.1) | 0.135 |
| Prior bleeding | 7 (1.5) | 11 (3.2) | 3 (2.3) | 0.308 |
| Active neoplasm | 22 (4.8) | 22 (6.5) | 6 (4.6) | 0.763 |
| Total PEGASUS criteria | 2.5 (1) | 3.1 (1) | 3.2 (1) | <0.001 |
| High bleeding risk criteria (%) | 153 (33.6) | 247 (72.2) | 116 (88.5) | <0.001 |
| Creatinine clearance (mL/min) | 80 (23) | 59 (21) | 45 (14) | <0.001 |
| Clinical presentation ACS^c^   - Unstable angina - NSTEMI^d^ - STEMI^e^ | 74 (16.3)  172 (38)  207 (45.7) | 54 (15.9)  148 (43.7)  137 (40.4) | 28 (21.5)  58 (44.6)  44 (33.8) | 0.023 |
| Left main or multivessel disease | 310 (68.4) | 261 (76.5) | 105 (80.2) | 0.002 |
| Number of stents | 1.5 (1) | 1.7 (1) | 1.4 (1) | 0.447 |
| Total length stents (mm) | 31 (22) | 32 (22) | 29 (17) | 0.353 |
| Complete revascularisation | 116 (26.1) | 80 (23.5) | 32 (24.8) | 0.567 |
| Left ventricle ejection fraction (%) | 56 (10) | 55 (9) | 52 (11) | 0.026 |
| Killip class on admission ≥ II | 54 (12) | 57 (16.9) | 25 (19.4) | 0.015 |
| P2Y12 inhibitor at discharge  Clopidogrel  Ticagrelor  Prasugrel | 120 (26.4)  49 (10.8)  285 (62.8) | 175 (51.6)  4 (1.2)  160 (47.2) | 106 (80.9)  1 (0.8)  24 (18.3) | <0.001 |
| DAPT^f^ discontinuation   - Before 1 year - At 1 year - After 1 year | 29 (6.4)  279 (61.3)  147 (32.3) | 24 (7)  213 (62.3)  105 (30.7) | 19 (14.5)  66 (50.4)  46 (35.1) | 0.340 |

Categorical variables are expressed as n (%). Quantitative variables are expressed as mean (SD).

a) MI: myocardial infarction; b) PCI: percutaneous coronary intervention; c) ACS: acute coronary syndrome; d) NSTEMI: Non-ST-segment elevation myocardial infarction; e) STEMI: ST-segment elevation myocardial infarction; f) DAPT: dual antiplatelet therapy.
