# Supplementary figures and images for: Extended use of dual antiplatelet therapy among older adults with acute coronary syndromes and associated variables: a cohort study
Source: Thromb J. 2023 Mar 21;21:32. doi: 10.1186/s12959-023-00476-5 (PMC10031931; doi:10.1186/s12959-023-00476-5)

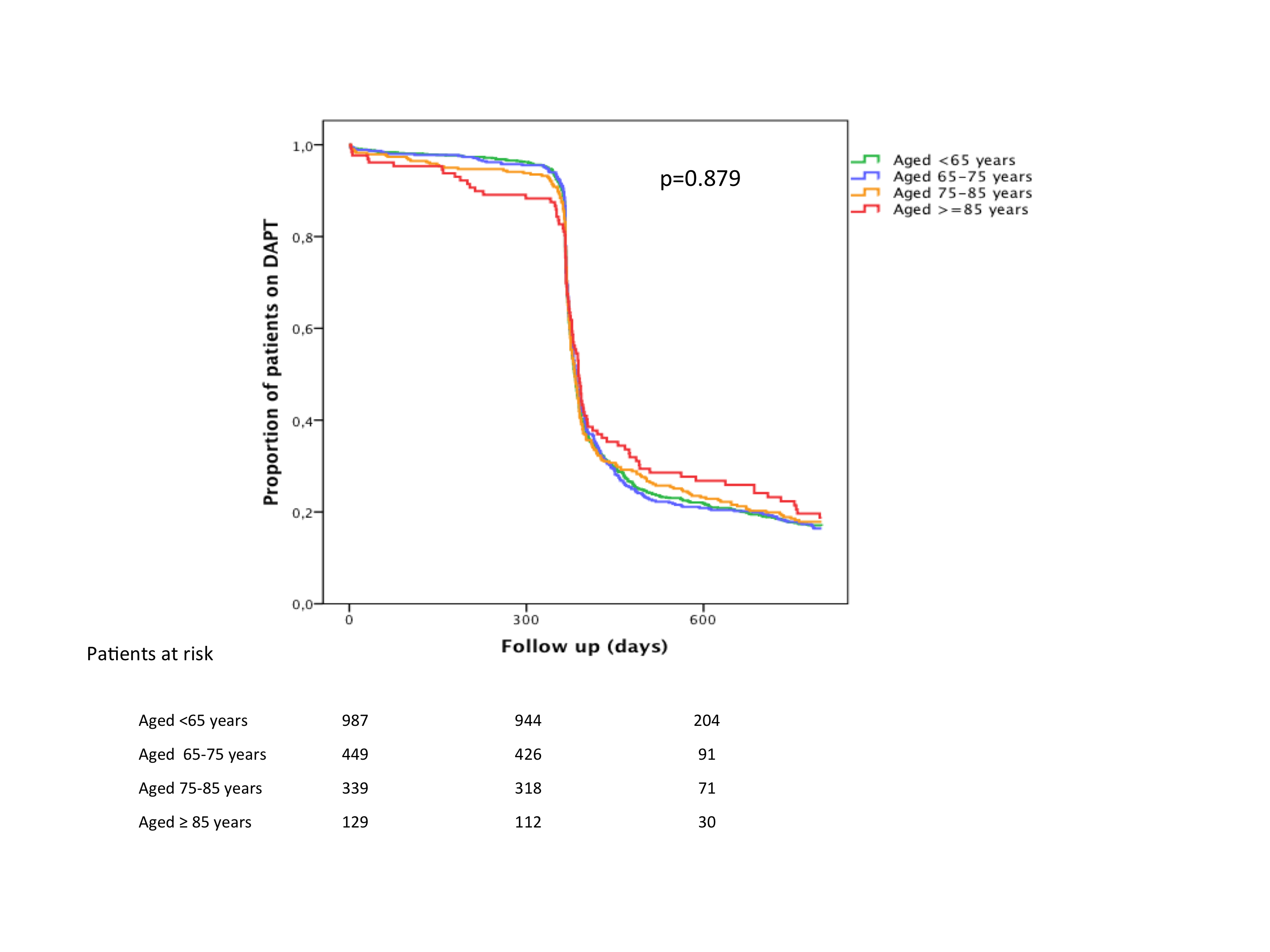

Supplement: Supplementary file 2 — Supplementary Material 2 [file 12959_2023_476_MOESM2_ESM.tif]
